# Air-Bubble-Insensitive Microfluidic Lactate Biosensor for Continuous Monitoring of Lactate in Sweat

**DOI:** 10.1021/acssensors.3c00490

**Published:** 2023-05-22

**Authors:** Isao Shitanda, Yuro Ozone, Yuki Morishita, Hiroyuki Matsui, Noya Loew, Masahiro Motosuke, Takahiro Mukaimoto, Momoko Kobayashi, Taketo Mitsuhara, Yamato Sugita, Kensuke Matsuo, Shinya Yanagita, Tatsunori Suzuki, Tsutomu Mikawa, Hikari Watanabe, Masayuki Itagaki

**Affiliations:** † Department of Pure and Applied Chemistry, Faculty of Science and Technology, Tokyo University of Science, 2641 Yamazaki, Noda 278-8510, Chiba, Japan; ‡ Research Institute for Science and Technology, Tokyo University of Science, 2641 Yamazaki, Noda 278-8510, Chiba, Japan; § Department of Mechanical Engineering, Faculty of Engineering, Tokyo University of Science, 6-3-1 Niijuku, Katsushika-ku, Tokyo 125-8585, Japan; ∥ Research Center for Organic Electronics (ROEL), Yamagata University, 4-3-16 Jonan, Yonezawa 992-8510, Yamagata, Japan; ⊥ Institute of Arts and Sciences, Tokyo University of Science, 2641 Yamazaki, Noda 278-8510, Chiba, Japan; # Department of Pharmacy, Faculty of Pharmaceutical Sciences, Tokyo University of Science, 2641 Yamazaki, Noda 278-8510, Chiba, Japan; ¶ Department of Globe Fire Science and Technology, Faculty of Science and Technology, Tokyo University of Science, 2641 Yamazaki, Noda 278-8510, Chiba, Japan; ∇ RIKEN Center for Biosystems Dynamics Research, 1-7-22 Suehiro-cho, Tsurumi-ku, Yokohama 230-0045, Kanagawa, Japan

**Keywords:** lactate biosensor, continuous monitoring, microfluidics, sweat analysis, wearable sensor

## Abstract

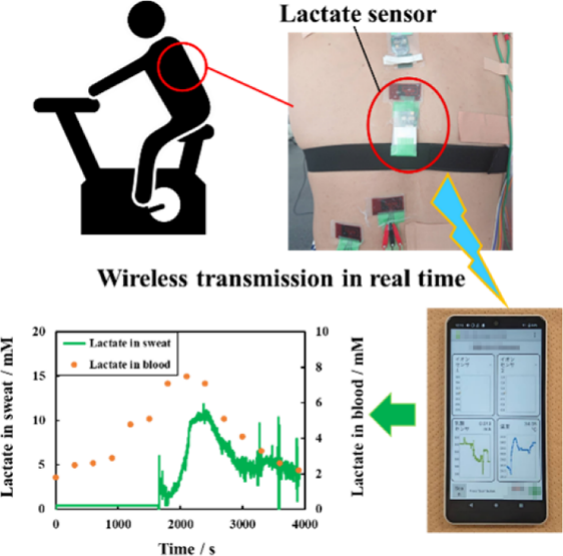

This study aimed to develop a lactate sensor with a microchannel that overcomes the issue of air bubbles interfering with the measurement of lactate levels in sweat and to evaluate its potential for continuous monitoring of lactate in sweat. To achieve continuous monitoring of lactate, a microchannel was used to supply and drain sweat from the electrodes of the lactate sensor. A lactate sensor was then developed with a microchannel that has an area specifically designed to trap air bubbles and prevent them from contacting the electrode. The sensor was evaluated by a person while exercising to test its effectiveness in monitoring lactate in sweat and its correlation with blood lactate levels. Furthermore, the lactate sensor with a microchannel in this study can be worn on the body for a long time and is expected to be used for the continuous monitoring of lactate in sweat. The developed lactate sensor with a microchannel effectively prevented air bubbles from interfering with the measurement of lactate levels in sweat. The sensor showed a concentration correlation ranging from 1 to 50 mM and demonstrated a correlation between lactate in sweat and blood. Additionally, the lactate sensor with a microchannel in this study can be worn on the body for an extended period and is expected to be useful for the continuous monitoring of lactate in sweat, particularly in the fields of medicine and sports.

Wearable devices have garnered considerable attention in recent years, and their potential applications in the fields of sports, medicine, and nursing care are highly anticipated. Electrochemical biosensors have been developed to determine health conditions by monitoring biomarkers in body fluids.^1−5^ Among these biomarkers, the lactate level is an important indicator of anaerobic metabolism.^6^ Under aerobic conditions, such as during light exercise, glucose, oxygen, and the energy source for muscle contraction are always present, and glucose is broken down into pyruvate and eventually into carbon dioxide and water.^6^ However, under anaerobic conditions, such as during high-intensity exercise, the supply of oxygen is insufficient to break down the pyruvate produced and, as a result, lactate is generated from pyruvate. This process leads to the accumulation of lactic acid, which is then released into the blood and subsequently secreted as sweat. Therefore, lactate levels in both blood and sweat can be considered indicators of exercise and are expected to be utilized in athlete training.^6^

Electrochemical biosensors are advantageous owing to their low-cost, small size, light weight, and their ability to be mass-produced, making them a viable option for quantifying lactate levels in body fluids. Previous studies have reported the development of lactate sensors that use lactate oxidase (LOx) and a mediator, an electron-transfer substance.^7−9^ The sensor works by transporting electrons generated by the enzymatic reaction between LOx and lactate to the electrode through a mediator and detecting the current. LOx acts as a catalyst that facilitates biological reactions; furthermore, it has substrate specificity for identifying certain molecules and reaction selectivity that allows for the selective quantification of lactate as a substrate.

Currently, the standard approach for athletic training involves measuring blood lactate levels before and after exercise.^10−12^ However, this method can place a significant burden on the subject’s body and is not conducive to continuously monitoring lactate concentration. Consequently, research has been conducted to develop biosensors that can continuously monitor lactate levels noninvasively using sweat, which can be easily collected, as a measurement solution.^13,14^ This approach eliminates the need for blood sampling and minimizes stress or discomfort to the subject while providing an opportunity to determine their health status.

Continuous monitoring of lactate in sweat requires a system that supplies and discharges sweat to the electrodes of a lactate sensor. Consequently, research has been conducted on developing a lactate sensor with microfluidic channels that can supply and discharge solutions.^13,15−19^ Microfluidics are created using microfabrication technology to form microfluidic channels of various shapes on substrates such as polydimethylsiloxane (PDMS), glass, and paper.^20^ This processing technology can operate at the nanometer to millimeter scale, allowing for the analysis of minute amounts of sample volumes. Additionally, because the analysis can be conducted in a confined space, instead of a large analyzer as in the past, it can be used as a wearable device for detecting biomarkers in sweat. For instance, Wang et al. developed a device that combines flexible PDMS-based microfluidics with a lactate sensor,^13^ which is highly wearable on the body. Similarly, Xiao et al. developed a device that integrates yarn/paper-based microfluidics with lactate and pH sensors.^15^ The device employs a hydrophilic thread that enables a smooth supply of even small amounts of sweat to the electrodes.

We have previously developed a device that integrated a lactate sensor with PDMS-based microfluidics.^21^ The soft and skin-friendly nature of PDMS made the device comfortable to wear for extended periods of time and adaptable to curved body parts. With a dynamic range of up to 50 mM, the sensor was observed to be capable of detecting the lactate threshold, indicating the metabolic shift from aerobic to anaerobic. However, a common issue of microfluidic channels is their tendency to trap air bubbles.^22−25^ If these air bubbles cover the electrodes of the sensor, the sensor response becomes unstable, interfering with the continuous monitoring of lactate.

In this study, a lactate sensor was developed that is unaffected by air bubbles infiltrating the PDMS channel. By introducing an air-trapping zone within the channel, the adverse effects of air bubbles on the sensor response were mitigated. The performance of the sensor was evaluated by examining its response to air bubbles and the solution flow rate and by attaching it to a person during exercise.

## Experimental Section

### Materials

LOx was prepared according to the method outlined in a previous study.^26^ Lactic acid, sodium lactate, and chitosan were purchased from Sigma-Aldrich. Thionine acetate, acetic acid, glycidyl methacrylate (GMA), *N,N*-dimethylformamide (DMF), 1-methyl-2-pyrrolidone (NMP), and dimethyl sulfoxide (DMSO) were purchased from Wako Pure Chemical Industries (Japan). MgOC (CNovel, average pore size 100 nm) was purchased from Toyo Tanso (Japan). Silver ink (SAP-40FL) and carbon ink (JELCON CH-8) were purchased from Sanwa Kagaku Kogyo (Osaka, Japan) and Jujo Chemical Co., Ltd. (Japan), respectively. Resist ink (S-40 C518) was obtained from TAIYO INK, while polyvinylidene fluoride hexafluoropropylene copolymer (PVDF #9305) was purchased from Kureha Corporation (Japan). Double-sided adhesive tape (4377N-50) was purchased from 3M Japan Ltd., while the silicone sheets were sourced from Tokawa Rubber Co (Japan). The hydrophilic agent (LAMBIC) was purchased from Osaka Organic Chemical Industry Co.

### Graft Polymerization of MgO-Templated Carbon

Grafted MgO-templated carbon (GMgOC) was prepared as described previously.^21,26^ Specifically, epoxy groups were introduced into the MgO-templated carbon (MgOC) using electron beam graft polymerization, allowing for stable immobilization of amino group-containing enzymes and mediators. The graft polymerization was carried out in DMF with 20 vol % GMA at 100 °C. The resulting GMgOC was washed with DMF and dried at 60 °C for 24 h. The grafting ratio of GMgOC was determined using thermogravimetric analysis (TGA, DSC1 Star System, Mettler-Toledo) and found to be 7.14%.

### Fabrication of the Screen-Printed Electrode

**Figure 1:**
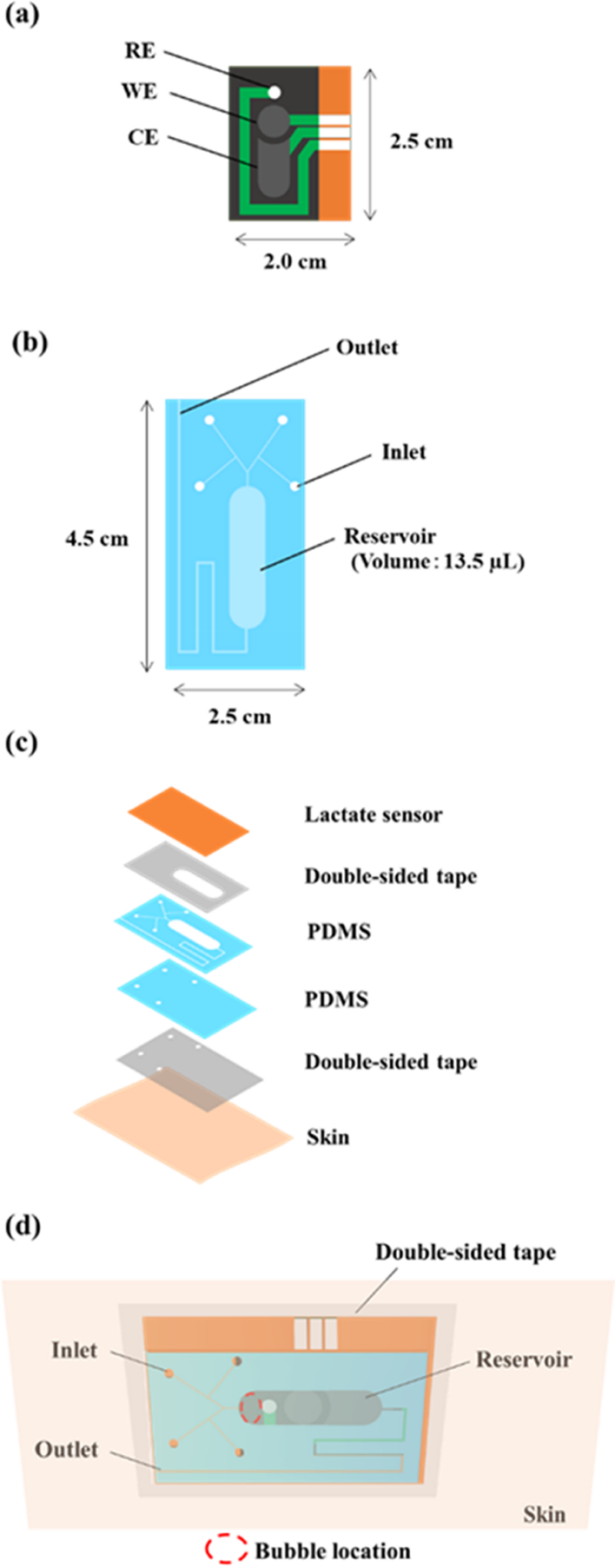
Design of lactate sensor and microfluidics. (a) 3-electrode chip. (b) Microfluidics. (c) Stacking diagram of a lactate sensor with microfluidics. (d) Diagram of a lactate sensor with flow path on skin.

Printed electrodes were fabricated on polyimide (PI) films using a screen printer (LS-150TV, NEWLONG SEIMITSU KOGYO CO. LTD., Japan) (Figure 1a). Three layers of silver ink were printed and dried at 130 °C for 30 min each to serve as leads. Working and counter electrodes were fabricated by printing five layers of carbon ink and drying at 120 °C for 30 min each, with the working electrode having an area of 0.196 cm^2^. An Ag/AgCl ink reference electrode was prepared by mixing Ag ink and AgCl in a weight ratio of 10:1. The resist layer was then printed and dried at 180 °C for 1 h. GMgOC ink was printed onto the working electrode and dried at 60 °C for 24 h. The ink was prepared by mixing GMgOC with PVDF (3.5 mL/1 g carbon) and NMP (6.0 mL/1 g carbon) until a smooth paste was obtained.

### Fabrication of the Screen-Printed Lactate Biosensor

2.5 μL of 50 mM thionine solution in methanol was deposited on the working electrode and allowed to dry for 30 min at room temperature. Next, 10 μL (15 U) of LOx solution (LOx, 50 U/mg) in 10 mM phosphate buffer (PB) (pH = 7.0) was dropped onto the dry mediator-modified working electrode and allowed to dry under reduced pressure for 2.5 h. Finally, 3.0 μL of chitosan-genipin solution was dropped onto the working electrode before drying in a refrigerator (4 °C) for two nights. To prepare the chitosan-genipin solution, chitosan (12 mg/mL) was dissolved in 0.5% (v/v) acetic acid solution and stirred (500 rpm) at 40 °C for 2 h. Next, 6 mg/mL genipin solution in DMSO was added to the chitosan solution, and the mixture was stirred for another 30 min.

### Fabrication of the Microfluidics

The microfluidic channels were fabricated on silicon sheets using a laser-processor (HAZIME CLI PLUS, Oh-Laser). The design is shown in Figure 1b. The fabricated channels were treated with UV ozone (PL16-110, SEN LIGHTS CORPORATION) for 15 min for cleaning and modification, followed by applying a drop of LAMBIC hydrophilic agent and drying at 80 °C for 20 min. The treated microfluidics were affixed to the lactate sensor using a 50 μm thick double-sided tape (4377N-50), as depicted in Figure 1c.

### Electrochemical Evaluation

The fabricated lactate sensor was evaluated via chronoamperometry (CA) using a potentiostat (EmStat3; Palm Sens). The measurements were conducted in a 0.1 M phosphate buffer solution with lactate concentrations ranging from 0 to 50 mM. For chronoamperometry, a potential of +0.1 V vs Ag/AgCl was applied.

Artificial sweat glands, which are a microfluidic system that divides a solution into four streams, were fabricated on a silicon wafer using photoresist. A lactate sensor with a channel was affixed to the artificial sweat gland using double-sided tape, and lactate was supplied through the four inlets of the channel. The solution was delivered using a syringe pump (Pump 11 Elite syringe pump, Harvard Apparatus) (Figure S1).

## Results and Discussion

### Design of the Microfluidic System

When a lactate sensor with a microfluidic system is attached to a person who is exercising, air bubbles may infiltrate the channel during measurement. Once inside the system, these air bubbles often stay trapped as fluid flows around them. In this study, a microfluidic system was designed that minimizes the influence of such air bubbles. Figure 1d depicts the attachment of the lactate sensor equipped with microfluidics to the skin. The front view of the figure showcases the skin, double-sided tape, and lactate sensor with the channel. When perspiration starts, sweat infiltrates through the four inlets of the channel via pressure and capillary action and is conveyed to the location of the electrode (i.e., the reservoir) on the lactate. The system operates in such a manner that old sweat is expelled through the outlet while new sweat enters. By increasing the length of the reservoir in the channel, a space was created to entrap air bubbles that had infiltrated, thereby preventing them from contacting the electrodes of the sensor. The volume of the bubble-trapping region in the channel was approximately 4.0 μL, which exceeds the volume of bubbles that entered during the on-body test. The thickness of the channel’s reservoir, including the 50 μm thickness of the double-sided tape, was determined to be 150 μm to reduce the response time of the lactate sensor (Figure S2). A layer of sweatproof double-sided tape with a larger area than the channel was inserted between the sensor with the channel and the skin to prevent sweat from flowing back from the outlet and entering the channel.

### Influence of Bubbles in Microchannels

**Figure 2:**
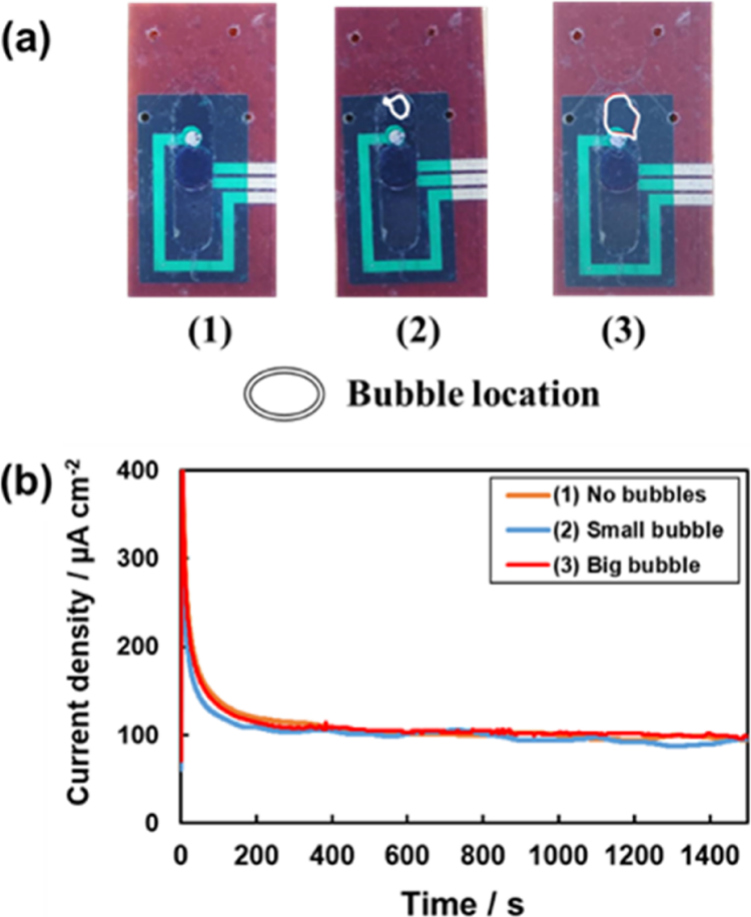
Lactate sensor with microfluidic channels in 0.1 M phosphate buffer (pH 4.5) containing 25 mM lactate at 10 μL/min flow rate in the presence of (1) no bubbles, (2) small bubbles, and (3) big bubbles. (a) Photos of sensors and microfluidics with bubbles. (b) Current response of sensor.

The impact of air bubbles entering the microfluidic system on the response of the lactate sensor was investigated (Figure 2). The measurements were performed using chronoamperometry for three cases, namely (1) no bubbles, (2) small bubbles, and (3) large bubbles. A phosphate buffer solution containing 25 mM lactate served as the measurement solution, and the solution was delivered into the channel at a flow rate of 10 μL/min using a syringe pump. As illustrated in Figure 2, no current value discrepancies were observed between cases with and without bubbles in the channel. This outcome suggests that the response of the sensor remained unaffected by bubbles, even if they entered the microfluidic system during measurement.

### Influence of the Flow Rate of Solution

**Figure 3:**
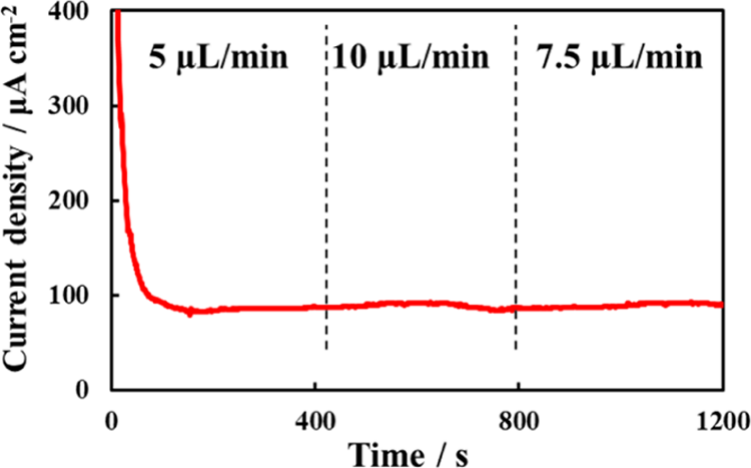
Response of lactate sensor with microfluidics to changing flow rate in 0.1 M phosphate buffer (pH 4.5) containing 25 mM lactate.

When monitoring lactate levels in sweat, changes in the amount of perspiration may occur due to changes in temperature and exercise intensity. Therefore, the effect of solution flow rate on the response of the lactate sensor with a flow channel was investigated using chronoamperometry (Figure 3). A phosphate buffer solution containing 20 mM lactate was used for measurement. The flow rate of the solution pumped from the syringe pump was set to 5–10 μL/min based on the results of the sweat rates of a person exercising. Although the flow rate of the solution changed during the measurements, no significant difference was observed in the current. This can be attributed to the diffusion of lactate between the chitosan membrane and the LOx layer being rate-limiting because the chitosan membrane on the electrode of the lactate sensor restricts the supply of the substrate lactate.^14,27^ Therefore, we conclude that the flow rate of the solution, that is, the amount of perspiration, had little influence on the response of the lactate sensor.

### Evaluation of Concentration Correlation

When monitoring lactate levels in sweat, the response of the lactate sensor must remain stable for several hours. To evaluate the stability of the sensor, chronoamperometry was performed for approximately 2 h using the fabricated lactate sensor with microfluidics (Figure S3). The measurement solution used was 0.1 M phosphate buffer containing 20 mM lactate, with a flow rate of 10 μL/min pumped from the syringe pump. The lactate sensor response remained stable for approximately 2 h after the start of the measurement, indicating successful immobilization of LOx and thionine on the electrode surface using GMgOC and chitosan membranes.

**Figure 4:**
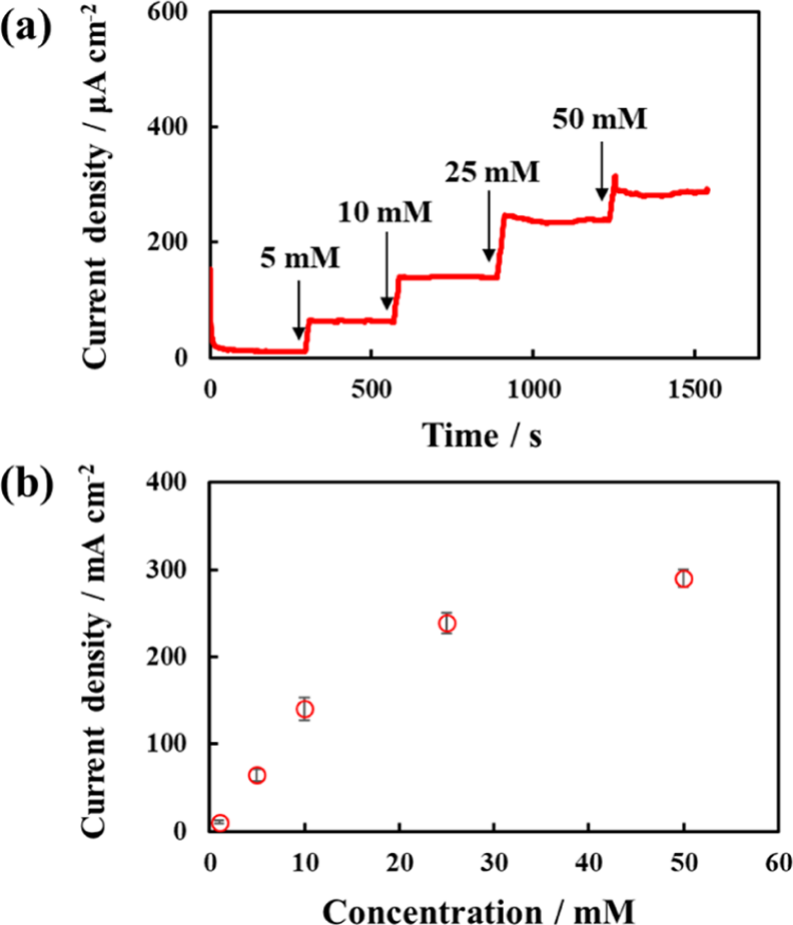
(a) Responses and (b) corresponding calibration curves of lactate sensor with microfluidic system in 0.1 M phosphate buffer (pH 7.5) containing various amounts of lactate at 10 μL/min flow rate. Error bars are the standard deviation with *n* = 4.

To test the sensor’s sensitivity to lactate concentration, a phosphate buffer solution containing various concentrations of lactate was pumped into the channel using a syringe pump, and the sensor’s response to increasing lactate concentration was recorded (Figure 4a). The measurement solution used was phosphate buffer (pH 7.5, 35 °C) containing 1–50 mM lactate, with a flow rate of 10 μL/min from the syringe pump. The measurements were conducted four times, and the current density was observed to increase with increasing lactate concentration. The time required for the current to stabilize with changes in lactate concentration was approximately 60 s, which is the approximate estimate of the response time during measurement.

A calibration curve was plotted, as shown in Figure 4b, to represent the correlation between lactate concentration and current. The results confirmed the correlation of lactate concentrations within the range of 1–50 mM lactate. The current increased linearly with lactate concentration within the range of 1–10 mM lactate and non-linearly at higher lactate concentrations. The sensitivity of the sensor in the linear range was calculated to be 14.5 μA/cm^2^/mM. Because lactate in sweat can reach up to approximately 50 mM,^28,29^ these results suggest that the lactate sensor can be used to monitor lactate levels in sweat.

### On-Body Test

**Figure 5:**
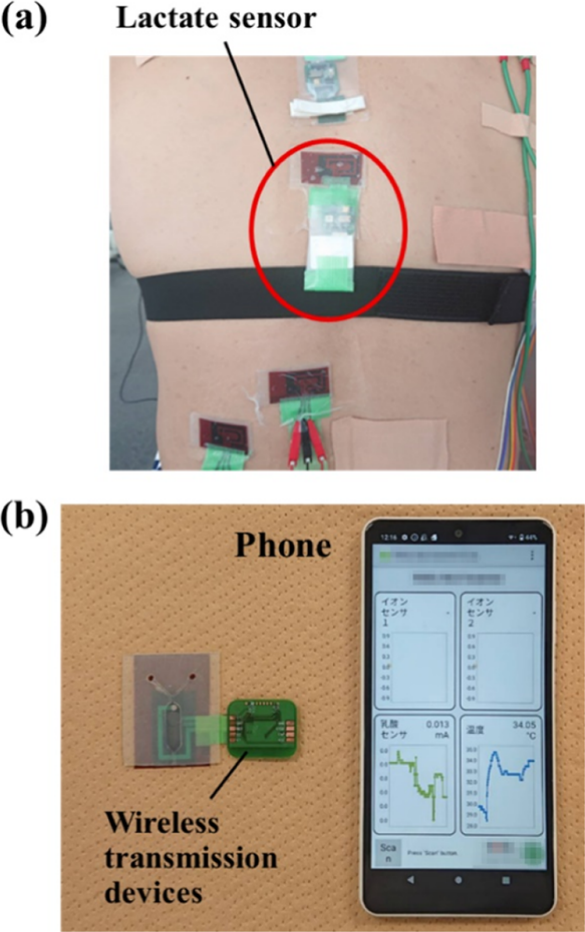
(a) Lactate sensor with microfluidics attached to the back of the test subject. (b) Monitoring lactate using wireless transmission devices and lactate sensors with microfluidic system.

Lactate sensors with microfluidics were tested on a male subject in his 40s who exercised on an aerobike. The lactate sensor was attached to the back of the participant (Figure 5a), and a wireless transmission device was used to monitor lactate in sweat wirelessly (Figure 5b).

Blood lactate levels were measured using a commercially available lactate sensor (Lactate Pro2, Arkray Inc.) to investigate the correlation between lactate levels in sweat and blood. Because the pH of sweat and body temperature vary among subjects^30,31^ and influence the activity of the enzyme LOx,^32^ pH and temperature were measured using a pH meter (LAQUAtwin, HORIBA) and a wearable thermometer (Vaitalgram CT2, AffordSENS), respectively.

**Figure 6:**
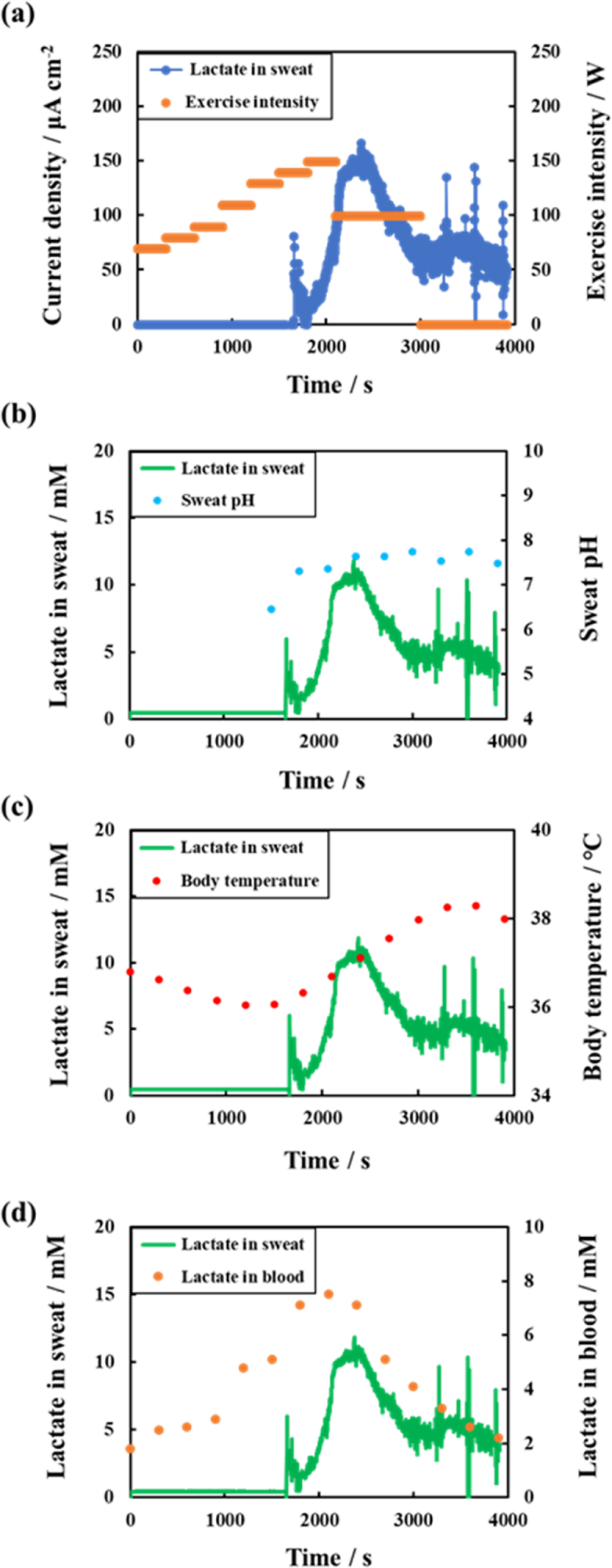
Lactate concentration in sweat determined using a lactate sensor with microfluidics during on-body test compared to other parameters: (a) exercise intensity; (b) sweat pH; (c) body temperature; and (d) blood lactate level.

Figure 6a depicts the current values obtained from the fabricated lactate sensor, alongside the results of the exercise load on the aerobike. The current value increased as the exercise load intensified, resulting in perspiration and sweat entering the channel, which was then supplied to the electrode after approximately 1600 s from the start of the measurement. The lactate concentration in sweat was observed to increase with the exercise load and current. Figure 6b illustrates the lactate concentration in sweat and sweat pH. The lactate concentration in sweat was converted into the lactate concentration from the current value using a calibration curve (Figure 4b). The pH of the subject’s sweat remained approximately 7.5 during the implementation test and was independent of the exercise load. The subject’s body temperatures were recorded between 36 and 38 °C. Figure 6d demonstrates the lactate levels in sweat and blood. Lactate concentrations in sweat and blood were observed to increase initially and then decrease, indicating a correlation between sweat lactate and blood lactate levels. These findings align with the previous studies that have reported a correlation between sweat and blood lactate levels.^33,34^ These results suggest that a lactate sensor with microfluidic channels can be used to monitor lactate in sweat.

For accurate determination of lactate concentration in human sweat using the sensing system developed here, calibration must account for temperature and pH. Of the two influencing parameters, temperature is less significant as human body temperature should not vary sufficiently to significantly influence the response current of the lactate sensor. The pH value of human sweat, however, can vary sufficiently and needs to be compensated for. During the on-body test shown in this study, pH did not vary significantly (Figure 6b), and the current values could be converted into lactate concentration values using the calibration curve in Figure 4b. However, other on-body tests required other calibration curves (data not shown). In the future, an integrated pH sensor would be desirable.

## Conclusions

In this study, a lactate sensor with a microfluidic system was developed to continuously monitor lactate in sweat. Previously reported lactate sensors with microfluidics have exhibited instability of response when air bubbles in the channel come into contact with the electrode. To address this issue, a reservoir with a designated area for trapping air bubbles was created to prevent contact with the electrode. Measurements were performed with and without air bubbles in the channel, and no difference was observed in the response of the lactate sensor. An on-body test was conducted using the fabricated sensor on a human subject, and a similar trend was observed between lactate levels in sweat and blood. As the microfluidics were fabricated from a soft, flexible, and non-irritating material, the lactate sensor developed in this study can be used to continuously monitor lactate in sweat, and its potential application in sports and medicine is promising.

## Supporting Information Available

Measurement methods, relationship between the lactate sensor response time and reservoir thickness, and responses of the lactate sensor with microfluidics (PDF)

## References

[ref1] Yang Y.; Gao W. Wearable and Flexible Electronics for Continuous Molecular Monitoring. Chem. Soc. Rev. 2019, 48, 1465–1491. 10.1039/C7CS00730B. 29611861

[ref2] Zheng X.; Zhang F.; Wang K.; Zhang W.; Li Y.; Sun Y.; Sun X.; Li C.; Dong B.; Wang L.; Xu L. Smart Biosensors and Intelligent Devices for Salivary Biomarker Detection. TrAC Trends Anal. Chem. 2021, 140, 116281 10.1016/j.trac.2021.116281.

[ref3] Takeda K.; Kusuoka R.; Inukai M.; Igarashi K.; Ohno H.; Nakamura N. An Amperometric Biosensor of L-Fucose in Urine for the First Screening Test of Cancer. Biosens. Bioelectron. 2021, 174, 112831 10.1016/j.bios.2020.112831. 33288426

[ref4] Wang J.; Wang L.; Li G.; Yan D.; Liu C.; Xu T.; Zhang X. Ultra-small Wearable Flexible Biosensor for Continuous Sweat Analysis. ACS Sens. 2022, 7, 3102–3107. 10.1021/acssensors.2c01533. 36218347

[ref5] Parmar J.; Patel S. K. Tunable and Highly Sensitive Graphene-Based Biosensor with Circle/Split Ring Resonator Metasurface for Sensing Hemoglobin/Urine Biomolecules. Phys. B 2022, 624, 413399 10.1016/j.physb.2021.413399.

[ref6] Jia W.; Bandodkar A. J.; Valdés-Ramírez G.; Windmiller J. R.; Yang Z.; Ramírez J.; Chan G.; Wang J. Electrochemical Tattoo Biosensors for Real-Time Noninvasive Lactate Monitoring in Human Perspiration. Anal. Chem. 2013, 85, 6553–6560. 10.1021/ac401573r. 23815621

[ref7] Payne M. E.; Zamarayeva A.; Pister V. I.; Yamamoto N. A. D.; Arias A. C. Printed, Flexible Lactate Sensors: Design Considerations Before Performing On-Body Measurements. Sci. Rep. 2019, 9, 13720 10.1038/s41598-019-49689-7. 31548553 PMC6757068

[ref8] Bollella P.; Sharma S.; Cass A. E. G.; Antiochia R. Minimally Invasive Microneedle-Based Biosensor Array for Simultaneous Lactate and Glucose Monitoring in Artificial Interstitial Fluid. Electroanalysis 2019, 31, 374–382. 10.1002/elan.201800630.

[ref9] Tuteja S. K.; Ormsby C.; Neethirajan S. Noninvasive Label-Free Detection of Cortisol and Lactate Using Graphene Embedded Screen-Printed Electrode. Nano-Micro Lett. 2018, 10, 41 10.1007/s40820-018-0193-5. PMC6199085 30393690

[ref10] Goodwin M. L.; Harris J. E.; Hernández A.; Gladden L. B. Blood Lactate Measurements and Analysis During Exercise: A Guide for Clinicians. J. Diabetes Sci. Technol. 2007, 1, 558–569. 10.1177/193229680700100414. 19885119 PMC2769631

[ref11] Nikseresht A.; Yabande I.; Rahmanian K.; Jahromi A. S. Blood Lactate Level in Elite Boy Swimmers After Lactate Tolerance Exercise Test. Biomed. Res. Ther. 2017, 4, 1318–1326. 10.15419/bmrat.v4i05.170.

[ref12] Obmiński Z.; Lerczak K.; Witek K.; Pintera M. Studies on Lactate Peak in Blood Following Judo Match. J. Combat Sports Martial Arts. 2010, 1, 95–99.

[ref13] Martín A.; Kim J.; Kurniawan J. F.; Sempionatto J. R.; Moreto J. R.; Tang G.; Campbell A. S.; Shin A.; Lee M. Y.; Liu X.; Wang J. Epidermal Microfluidic Electrochemical Detection System: Enhanced Sweat Sampling and Metabolite Detection. ACS Sens. 2017, 2, 1860–1868. 10.1021/acssensors.7b00729. 29152973

[ref14] Xuan X.; Pérez-Rafols C.; Chen C.; Cuartero M.; Crespo G. A. Lactate Biosensing for Reliable On-Body Sweat Analysis. ACS Sens. 2021, 6, 2763–2771. 10.1021/acssensors.1c01009. 34228919 PMC8397467

[ref15] Xiao G.; He J.; Qiao Y.; Wang F.; Xia Q.; Wang X.; Yu L.; Lu Z.; Li C. M. Facile and Low-Cost Fabrication of a Thread/Paper-Based Wearable System for Simultaneous Detection of Lactate and pH in Human Sweat. Adv. Fiber Mater. 2020, 2, 265–278. 10.1007/s42765-020-00046-8.

[ref16] Choi J.; Kang D.; Han S.; Kim S. B.; Rogers J. A. Thin, Soft, Skin-Mounted Microfluidic Networks with Capillary Bursting Valves for Chrono-Sampling of Sweat. Adv. Healthc. Mater. 2017, 6, 1601355 10.1002/adhm.201601355. 28105745

[ref17] Rossini E. L.; Milani M. I.; Lima L. S.; Pezza H. R. Paper Microfluidic Device Using Carbon Dots to Detect Glucose and Lactate in Saliva Samples. Spectrochim. Acta Part Molecure Biomolecure Spectrosc. 2021, 248, 119285 10.1016/j.saa.2020.119285. 33310613

[ref18] Ji X.; Lau H. Y.; Ren X.; Peng B.; Zhai P.; Feng S. P.; Chan P. K. L. Highly Sensitive Metabolite Biosensor Based on Organic Electrochemical Transistor Integrated with Microfluidic Channel and Poly(N-Vinyl-2-Pyrrolidone)-Capped Platinum Nanoparticles. Adv. Mater. Technol. 2016, 1, 1600042 10.1002/admt.201600042.

[ref19] Promphet N.; Rattanawaleedirojn P.; Siralertmukul K.; Soatthiyanon N.; Potiyaraj P.; Thanawattano C.; Hinestroza J. P.; Rodthongkum N. Non-invasive Textile Based Colorimetric Sensor for the Simultaneous Detection of Sweat pH and Lactate. Talanta 2019, 192, 424–430. 10.1016/j.talanta.2018.09.086. 30348413

[ref20] Yang W.; Yu M.; Sun X.; Woolley A. T. Microdevices Integrating Affinity Columns and Capillary Electrophoresis for Multibiomarker Analysis in Human Serum. Lab Chip 2010, 10, 2527–2533. 10.1039/C005288D. 20664867 PMC2998056

[ref21] Shitanda I.; Mitsumoto M.; Loew N.; Yoshihara Y.; Watanabe H.; Mikawa T.; Tsujimura S.; Itagaki M.; Motosuke M. Continuous Sweat Lactate Monitoring System with Integrated Screen-Printed MgO-Templated Carbon-Lactate Oxidase Biosensor and Microfluidic Sweat Collector. Electrochim. Acta 2021, 368, 137620 10.1016/j.electacta.2020.137620.

[ref22] Kang J. H.; Kim Y. C.; Park J. K. Analysis of Pressure-Driven Air Bubble Elimination in a Microfluidic Device. Lab Chip 2008, 8, 176–178. 10.1039/B712672G. 18094777

[ref23] Xu J.; Vaillant R.; Attinger D. Use of a Porous Membrane for Gas Bubble Removal in Microfluidic Channels: Physical Mechanisms and Design Criteria. Microfluid. Nanofluid. 2010, 9, 765–772. 10.1007/s10404-010-0592-5.

[ref24] Sung J. H.; Shuler M. L. Prevention of Air Bubble Formation in a Microfluidic Perfusion Cell Culture System Using a Microscale Bubble Trap. Biomed. Microdevices. 2009, 11, 731–738. 10.1007/s10544-009-9286-8. 19212816

[ref25] Nakayama T.; Hiep H. M.; Furui S.; Yonezawa Y.; Saito M.; Takamura Y.; Tamiya E. An Optimal Design Method for Preventing Air Bubbles in High-Temperature Microfluidic Devices. Anal. Bioanal. Chem. 2010, 396, 457–464. 10.1007/s00216-009-3160-7. 19841913

[ref26] Shitanda I.; Takamatsu K.; Niiyama A.; Mikawa T.; Hoshi Y.; Itagaki M.; Tsujimura S. High-Power Lactate/O2 Enzymatic Biofuel Cell Based on Carbon Cloth Electrodes Modified with MgO-Templated Carbon. J. Power Sources 2019, 436, 226844 10.1016/j.jpowsour.2019.226844.

[ref27] Tur-García E. L.; Davis F.; Collyer S. D.; Holmes J. L.; Barr H.; Higson S. P. J. Novel Flexible Enzyme Laminate-Based Sensor for Analysis of Lactate in Sweat. Sens. Actuators B. 2017, 242, 502–510. 10.1016/j.snb.2016.11.040.

[ref28] Derbyshire P. J.; Barr H.; Davis F.; Higson S. P. J. Lactate in Human Sweat: A Critical Review of Research to the Present Day. J. Physiol. Sci. 2012, 62, 429–440. 10.1007/s12576-012-0213-z. 22678934 PMC10717375

[ref29] Sakharov D. A.; Shkurnikov M. U.; Vagin M. Yu.; Yashina E. I.; Karyakin A. A.; Tonevitsky A. G. Relationship Between Lactate Concentrations in Active Muscle Sweat and Whole Blood. Bull. Exp. Biol. Med. 2010, 150, 83–85. 10.1007/s10517-010-1075-0. 21161059

[ref30] Sonner Z.; Wilder E.; Heikenfeld J.; Kasting G.; Beyette F.; Swaile D.; Sherman F.; Joyce J.; Hagen J.; Kelley-Loughnane N.; Naik R. The Microfluidics of the Eccrine Sweat Gland, Including Biomarker Partitioning, Transport, and Biosensing Implications. Biomicrofluidics 2015, 9, 031301 10.1063/1.4921039. 26045728 PMC4433483

[ref31] Patterson M. J.; Galloway S. D. R.; Nimmo M. A. Variations in Regional Sweat Composition in Normal Human Males. Exp. Physiol. 2000, 85, 869–875. 10.1017/S0958067000020583. 11187982

[ref32] Cunha-Silva H.; Pires F.; Dias-Cabral A. C.; Arcos-Martinez M. J. Inhibited Enzymatic Reaction of Crosslinked Lactate Oxidase Through a PH-Dependent Mechanism. Colloids Surf. B Biointerfaces 2019, 184, 110490 10.1016/j.colsurfb.2019.110490. 31536937

[ref33] Karpova E. V.; Laptev A. I.; Andreev E. A.; Karyakina E. E.; Karyakin A. A. Relationship Between Sweat and Blood Lactate Levels During Exhaustive Physical Exercise. ChemElectroChem 2020, 7, 191–194. 10.1002/celc.201901703.

[ref34] Seki Y.; Nakashima D.; Shiraishi Y.; Ryuzaki T.; Ikura H.; Miura K.; Suzuki M.; Watanabe T.; Nagura T.; Matsumato M.; Nakamura M.; Sato K.; Fukuda K.; Katsumata Y. A Novel Device for Detecting Anaerobic Threshold Using Sweat Lactate During Exercise. Sci. Rep. 2021, 11, 4929 10.1038/s41598-021-84381-9. 33654133 PMC7925537

