# Supplementary material for: Air-Bubble-Insensitive Microfluidic Lactate Biosensor for Continuous Monitoring of Lactate in Sweat
Source: ACS Sens. 2023 May 22;8(6):2368–74. doi: 10.1021/acssensors.3c00490 (PMC10294251; doi:10.1021/acssensors.3c00490)
Supplement: Supplementary file 1 — se3c00490_si_001.pdf [file se3c00490_si_001.pdf]

Supporting information

Air-bubble insensitive microfluidic lactate biosensor for continuous monitoring of lactate in sweat

Isao Shitanda,[1,2*] Yuro Ozone,[1‡] Yuki Morishita, [3‡] Hiroyuki Matsui,[4] Noya Loew,[1,‡] Masahiro Motosuke,[2,3] Takahiro Mukaimoto,[2,5] Momoko Kobayashi,[6] Taketo Mitsuhara,[7] Yamato Sugita,[7] Kensuke Matsuo,[7] Shinya Yanagita,[2,5] Tatsunori Suzuki,[6] Tsutomu Mikawa,[8] Hikari Watanabe,[1] and Masayuki Itagaki[1,2]

[1] Department of Pure and Applied Chemistry, Faculty of Science and Technology, Tokyo University of Science, 2641 Yamazaki, Noda, Chiba 278-8510, Japan

[2] Research Institute for Science and Technology, Tokyo University of Science, 2641 Yamazaki, Noda, Chiba 278-8510, Japan

[3] Department of Mechanical Engineering, Faculty of Engineering, Tokyo University of Science, 6-3-1 Niijuku, Katsushika-ku, Tokyo 125-8585, Japan

[4] Research Center for Organic Electronics (ROEL), Yamagata University, 4-3-16 Jonan, Yonezawa, Yamagata 992-8510, Japan

[5] Institute of Arts and Sciences, Tokyo University of Science, 2641 Yamazaki, Noda, Chiba 278-8510, Japan

[6] Department of Pharmacy, Faculty of Pharmaceutical Sciences, Tokyo University of Science, 2641 Yamazaki, Noda, Chiba 278-8510, Japan

[7] Department of Globe Fire Science and Technology, Faculty of Science and Technology, Tokyo University of Science, 2641 Yamazaki, Noda, Chiba 278-8510, Japan

[8] RIKEN Center for Biosystems Dynamics Research, 1-7-22 Suehiro-cho, Tsurumi-ku, Yokohama, Kanagawa 230-0045, Japan

‡These authors contributed equally

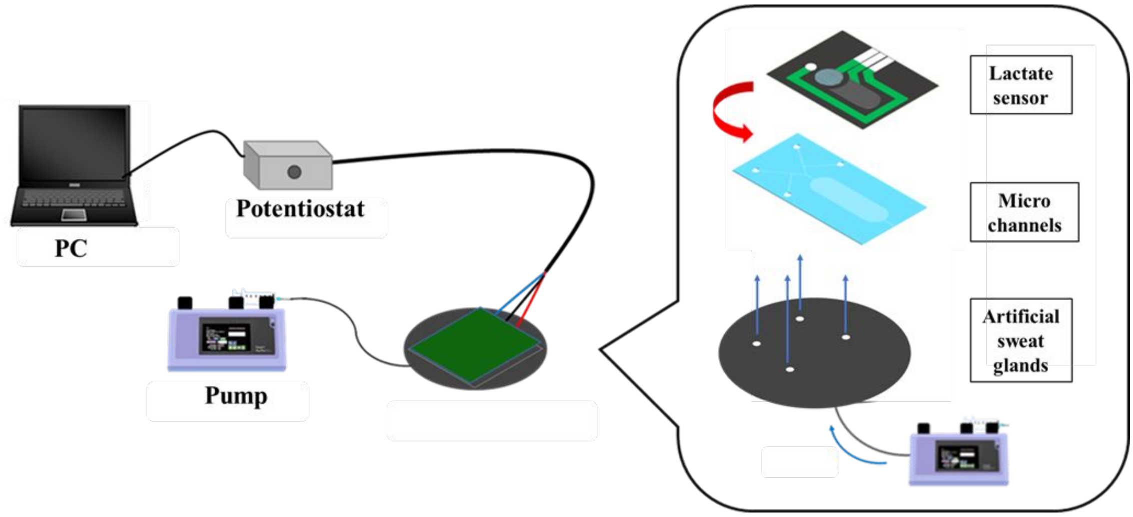

Figure S1. Measurement methods. A syringe pump was used to supply the solution to the microchannels through artificial sweat glands.

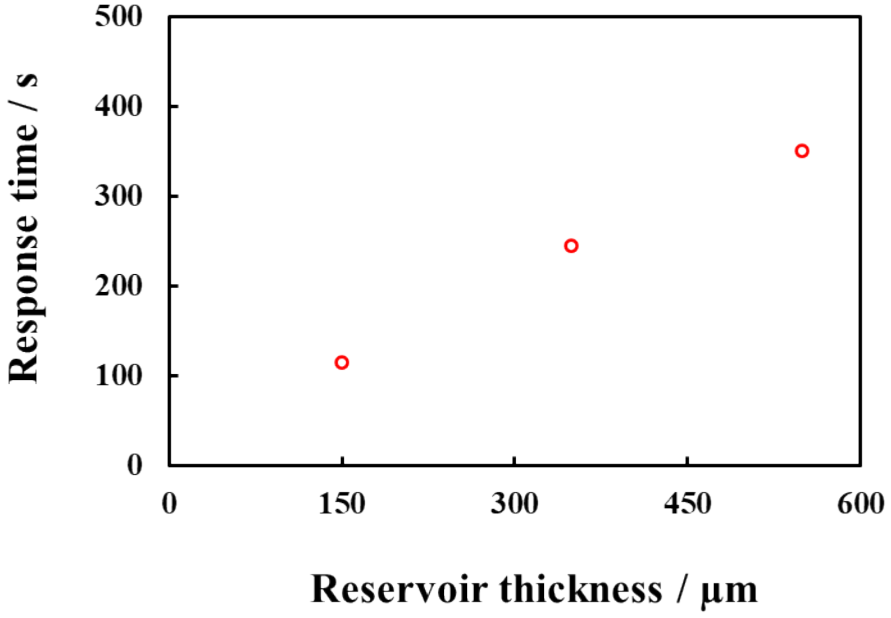

Figure S2. Relationship between lactate-sensor response time and reservoir thickness.

The response time is defined as the time required for the reservoir to fill the solution and stabilize the sensor response.

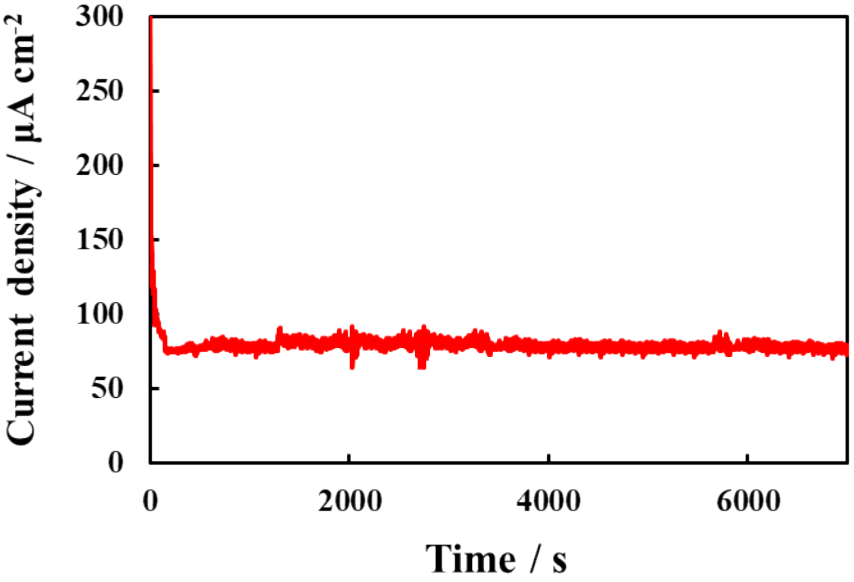

Figure S3. Responses of lactate sensor with microfluidics in 0.1 M phosphate buffer (pH 4.5) containing 20 mM lactate at 10 μL/min flow rate.
